# Identifying the genetic causes of phenotypically diagnosed Pakistani mucopolysaccharidoses patients by whole genome sequencing

**DOI:** 10.3389/fgene.2023.1128850

**Published:** 2023-04-05

**Authors:** Rutaba Gul, Sabika Firasat, Mikkel Schubert, Asmat Ullah, Elionora Peña, Anne C. B. Thuesen, Mulazim Hussain, Frederik F. Staeger, Anette P. Gjesing, Anders Albrechtsen, Torben Hansen

**Affiliations:** ^1^ Department of Zoology, Faculty of Biological Sciences, Quaid-I-Azam University, Islamabad, Pakistan; ^2^ Novo Nordisk Foundation Center for Basic Metabolic Research, Faculty of Health and Medical Sciences, University of Copenhagen, Copenhagen, Denmark; ^3^ The Children Hospital, Pakistan Institute of Medical Sciences (PIMS), Islamabad, Pakistan; ^4^ Section for Computational and RNA Biology, Department of Biology, University of Copenhagen, Copenhagen, Denmark

**Keywords:** lysosomal storage disorder, mucopolysaccharidosis, Pakistani families, whole genome sequencing, ABCA5

## Abstract

**Background:** Lysosomal storage disorders (LSDs) are a group of inherited metabolic diseases, which encompass more than 50 different subtypes of pathologies. These disorders are caused by defects in lysosomal enzymes, transporters, and other non-lysosomal proteins. Mucopolysaccharidosis (MPS) is the most common subgroup of lysosomal storage disorders in which the body is unable to properly breakdown mucopolysaccharides. The aim of the present study was to identify novel genes and pathogenic variants in families from diverse regions of Pakistan with clinically diagnosed mucopolysaccharidosis type I and mucopolysaccharidosis type II.

**Methods:** Clinical diagnosis identified 12 with mucopolysaccharidosis I and 2 with mucopolysaccharidosis II in 14 families and whole genome sequencing (WGS) was performed to identify the causative variations in 15 affected individuals. Twenty-two unaffected individuals including parents or normal siblings of patients were also sequenced. Putative causal variants were identified by co-segregation and functional annotation.

**Results:** Analysis of whole genome sequencing data revealed ten novel and six previously reported variants in lysosomal storage disorders-associated genes (*IDUA, GALNS, SGSH, GAA, IDS*, *ALDOB, TRAPPC4, MASP1, SMARCAL, KIAA1109, HERC1, RRAS2*) and a novel candidate gene (*ABCA5*) for lysosomal storage disorder-like phenotypes, which has previously been associated with symptoms strongly related with lysosomal storage disorder in animal models.

**Conclusion:** Multigenic inheritance was found in several families highlighting the importance of searching for homozygous pathogenic variants in several genes also in families with a high degree of consanguinity.

## 1 Introduction

Lysosomal storage disorders (LSDs) are a diverse group of inherited conditions primarily caused by loss of function of lysosomal proteins leading to the dysregulation of many lysosomes-linked activities including autophagy, lipid homeostasis, membrane repair, cell viability, exocytosis and signaling cascades (Parenti et al., 2015; Marques and Saftig, 2019). Common clinical phenotypes of LSDs include delay in achieving milestones, abdominal distention, hepatosplenomegaly, short stature, coarse facial features, and joint contractures (Gul et al., 2021). LSDs are classified into subtypes based on accumulation of undegraded substrates like glycosaminoglycans (GAGs), glycogens and sphingolipids accumulated as a result of compromised activities of enzymes in lysosomes.

Autosomal recessive disorders are more prevalent in populations like the Pakistani where 60% of all marriages are consanguineous and among them more than 80% are first cousin unions (Hussain and Bittles, 1998). Globally, the prevalence for LSDs ranges from 7.5/100,000 to 23.5/100,000 live births, with sphingolipidoses as the highest prevalent disorder followed by mucopolysaccharidoses (MPSs) (Kingma et al., 2015). Mucopolysaccharidoses (MPS) (OMIM # 252700) are carbohydrate metabolism disorders caused by the deficiency of lysosomal enzymes required for hydrolyzing glycosaminoglycans (GAGs; negatively charged polysaccharides). MPS are characterized by gradual accumulation of various types of GAGs within cells of multiple organs leading to somatic manifestations such as coarse facial features, cardiac issues, skeletal deformities, hepatosplenomegaly, respiratory problems, hematologic, neurologic, and ocular phenotypes. MPS include a spectrum of clinical phenotypes from severe to attenuated forms including all MPS sub-types like MPS I, MPS II, MPS III, MPS IV, MPS VI, MPS VII with overlapping physical features (Parenti and Giugliani, 2022). Most MPSs are inherited as autosomal recessive disorders except MPS II which follows an X-linked inheritance pattern (Linhart and Elliott, 2007).

Genetic defects in genes encoding lysosomal enzymes including *IDUA, IDS, SGSH, NAGLU, HGSNAT, GNS, GALNS, GLB1* and *GAA* lead to MPS. Additionally, pathogenic variants in other lysosomal proteins also lead to hereditary metabolic disorders with phenotypes that overlap with MPS (Kubo et al., 2005), indicating that other candidate genes may also cause or contribute to the disease. For example, ATP binding cassette (ABC) are group of transporters involved in transportation of substrates across membranes. *ABCA5* is a member of this group of transporters (Brooks-Wilson et al., 1999). ABCA5 is located in the lysosomes and late endosomes and its homozygous knockout results in lysosomal disease like symptoms in mice (Kubo et al., 2005).

Therapeutic management is a serious problem in LSDs as enzyme replacement therapy (ERT) has limitations including high cost, life-long treatment, inability to stop progression of neuronal symptoms and possibility of immune response against an exogenous enzyme (Arora et al., 2007). Globally, combinational therapies including ERT, and pharmacological chaperone therapy (PCT) are proving fruitful. For PCT, knowledge of the enzyme mutations is required for selection of a specific chaperone, reflecting the importance of identifying a molecular diagnosis. Other emerging treatment options including gene therapy, genome editing, and anti-inflammatory therapy also rely on identifying the genetic cause of the disease (Kingma and Jonckheere, 2021; Schuh et al., 2029). Early diagnosis for LSDs represents a big challenge and it is crucial to prevent multi-organ failure (Mokhtariye et al., 2019). The use of comprehensive genetic sequencing methodologies such as whole genome sequencing (WGS) are particularly suitable to elucidate the genetic background in rare diseases such as MPS I and MPS II, by enabling the detection of rare variants in known as well as novel genes. The aim of this study was to identify disease-causing variants using WGS data of 14 Pakistani families initially diagnosed as MPS I and MPS II based on clinical presentation.

## 2 Materials and methodology

### 2.1 Ethical approvals

Ethical approval was granted by the Bioethical Review board, Quaid-i-Azam University, Islamabad, Pakistan (BEC-FBS-QAU2019-198) and Shaheed Zulfiqar Ali Bhutto Medical University, Islamabad, Pakistan (F.1-1/2105/ERB/SZABMU/179). Before collection of blood samples, all participants or their guardians signed an informed consent form. The study was conducted in accordance with the Declaration of Helsinki (World Medical Association, 2013).

### 2.2 Participants

**FIGURE 1:**
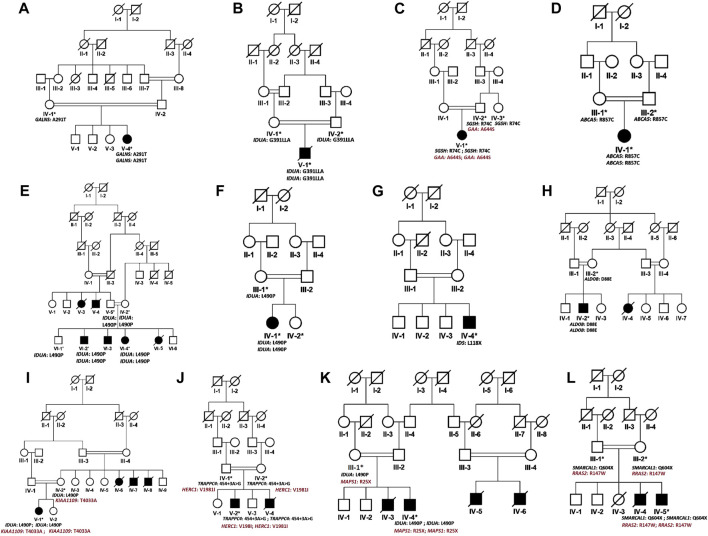
Pedigrees of families **(A–L)** inheriting MPS I phenotype consistent with autosomal recessive mode of inheritance of disease. Square and circles denote males and females respectively, filled symbols indicate affected individuals and consanguinity is represented by double marriage lines. Genotypes of participating individuals are shown beneath each individual.

**FIGURE 2:**
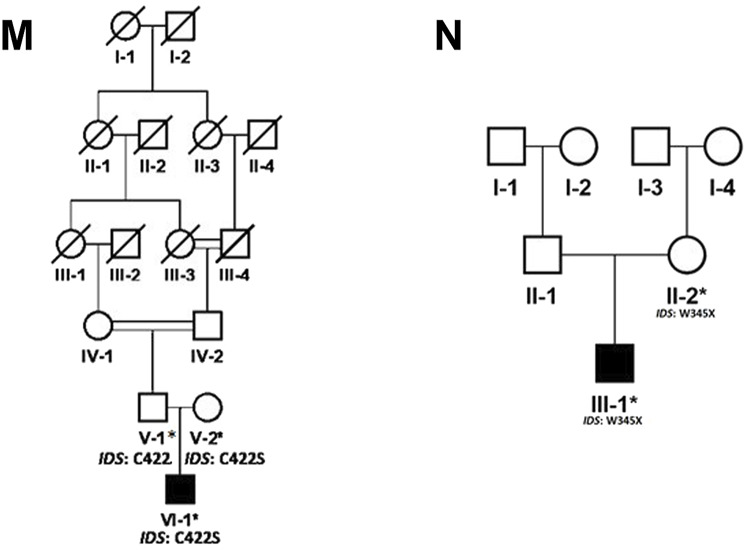
Pedigrees of families **(M,N)** inheriting MPS II phenotype consistent with X-linked mode of inheritance of disease. Square and circles denote males and females respectively, filled symbols indicate affected individuals and consanguinity is represented by double marriage lines. Genotypes of participating individuals are shown beneath each individual.

**FIGURE 3:**
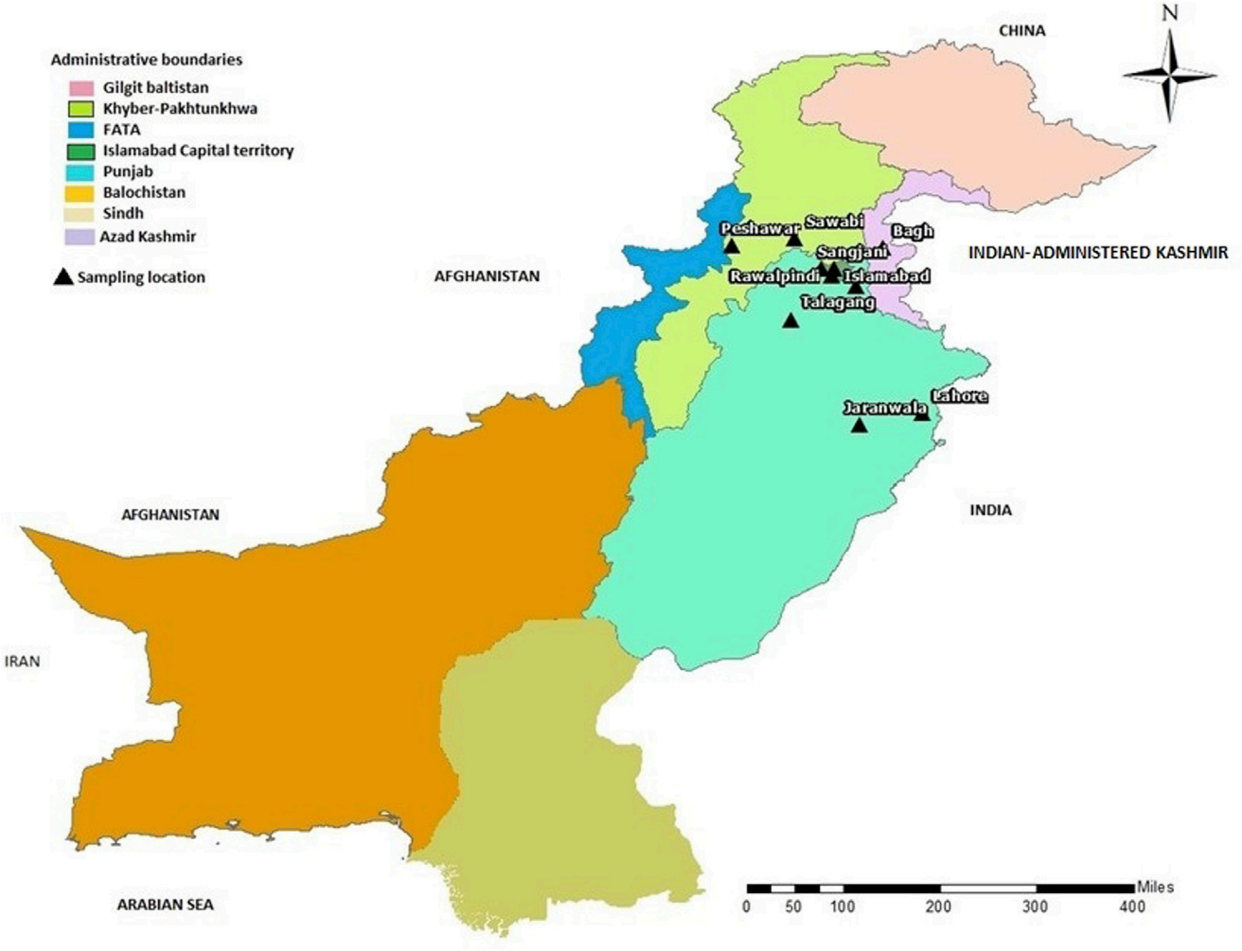
Map of Pakistan showing the locations (black triangles) of cities/towns from where families included in this study were collected.

In the present study, we recruited 14 families in total (A–N). Twelve of them (A–L) were diagnosed as MPS I with 26 affected individuals (Figure 1) and the other two (M, N) as MPS II with two affected males (Figure 2). In total, 37 individuals including 15 affected and 22 unaffected participated in the present study. Families were recruited from diverse regions of Pakistan (i.e., Punjab, Islamabad capital territory, Khyber Pakhtunkhwa, and Azad Jammu and Kashmir) (Figure 3). Moreover, 77 additional individuals with Pakistani origin that are part of an ongoing LSDs study (unpublished) were also sequenced.

Inclusion criteria were based on their physical phenotypes, radiological findings, and blood testing (Table 1) at outpatient department (OPD), Children Hospital, Pakistan Institute of Medical Sciences, Islamabad, Pakistan. Pedigrees were drawn by using the information provided by the accompanying elder attendant of the patients.

**TABLE 1:** Showing the details of each patient affected with Mucopolysaccharidosis type I and II.

Family ID	Individual no.	No. of affected individuals	Age/Sex	Cast/Region/Consanguinity	Clinical profile
A	V-4	1	9 m/F	Rajput/ICT/Yes	Squint eyes, tip-toe walking, kyphosis, frontal bossing, depressed nasal bridge, umbilical hernia, narrowing of spinal cord, dysostosis, throat problem, weak muscles, more crying episodes, two central incisors widely spaced, joint contractures, coarse facial features
					Pectus carinatum, Hemoglobin = 8.6 g/dL (12–15), HCT = 32 (35–55), MCV = 63.9 fl (75–100), MCH = 17.2 pg (25–35), MCHC = 26.9 g/dL (31–38), Neutrophils = 40.4% (60–70), ESR = 28 mm/Hr (0–15)
B	V-1	1	1 y/M	Malik/Punjab/Yes	Delay in achieving milestone, speech delay, hernia, short stature, coarse facial features, joint contractures, hepatosplenomegaly, developmental delay, depressed nasal bridge, squinted eyes
C	V-1	1	4 y/F	Pathan/ICT/Yes	Delay in achieving milestone, abdominal distention, aggressive behavior, speech delay, short stature, respiratory/ear infections, coarse facial features, broad based gait, joint contractures, short trunk, scoliosis/kyphosis, hepatosplenomegaly, low IQ, intellectual disability, low hemoglobin
D	IV-1	1	1.5 y/F	Sheikh/Punjab/Yes	Delay in achieving milestone, abdominal distention, aggressive behavior, speech delay, gibbus formation, short stature, upper airways obstruction, kyphosis, hazel corneas, coarse facial features, hearing loss, joint contractures, hepatosplenomegaly, Intellectual disability, hypertrichosis
E	VI-4	6	8 y/F	Rajput/AJK/Yes	Delay in achieving milestone, abdominal distention, aggressive behavior, speech delay, hernia, gibbus formation, short stature, respiratory/ear infection, coarse facial features, waddling in gait, joint contractures, scoliosis, hepatosplenomegaly, corneal clouding
	VI-2		3 y/M		Delay in achieving milestone, abdominal distention, aggressive behavior, speech delay, hernia, gibbus formation, short stature, respiratory/ear infection, coarse facial features, waddling in gait, joint contractures, scoliosis, hepatosplenomegaly, corneal clouding
F	IV-1	1	16.5 y/F	Narman/AJK/Yes	Delay in achieving milestones, abdominal distention, hernia, short stature, coarse facial features, joint contractures, short trunk, hepatosplenomegaly
G	IV-4	1	6 y/M	Rajput/Punjab/Yes	Delay in achieving milestones, abdominal distention, aggressive behavior, short stature, respiratory/ear infection, joint contractures, short trunk, speech delay, coarse facial features, hepatosplenomegaly, intellectual disability
H	IV-2	2	14 y/M	Suddhan/ICT/Yes	Delay in achieving milestones, abdominal distention, coarse facial features, aggressive behavior, short stature, respiratory/ear infections, hip dysplasia, joint stiffness, hepatosplenomegaly, difficulty in walking
					Craniofacial disproportion, J-shaped sella, L1 vertebral body is hypoplastic and slightly posteriorly displaced, exaggerated lumbar lordosis, RBG = 71 mg/dL (80–160), serum urea = 10 md/dL (12–50), loss of biconcavity of metacarpels with proximal tapping
I	V-1	4	12 y/F	Mughal/Punjab/Yes	Joint contractures, corneal clouding, very less eyesight, abdominal distention, coarse facial features, short stature, underweight, umbilical hernia, cannot walk, developmental delay, cardiac issues, hearing issues, respiratory problems/loud snoring
J	V-2	2	2.5 y/M	Mughal/KPK/Yes	Delay in achieving milestones, aggressive behavior with multiple crying episodes, respiratory/ear infection, no walk, intellectual disability
K	IV-4	4	8.5 y/M	Abbasi/Punjab/Yes	Delay in achieving milestones, abdominal distention, aggressive behavior, speech delay, umbilical hernia, gibbus formation, short stature, constipation, coarse facial features, cannot walk (Wheel chair bound), joint contractures, scoliosis and kyphosis, hepatosplenomegaly, fish mouth vertebrae, oar shaped ribs, low IQ, widely spaced eyes, narrowing of eye opening (right), drooping eyelids, intellectual disability, developmental delay
					Red cell count = 2.68 million/ul (4–5.2), Hemoglobin = 7.2 g/dL (11.5–15.5), HCT = 21.8 (35–45), Platelet count = 141 × 1000/ul (170–450), Neutrophils = 53.4% (60–70)
L	IV-5	2	8 y/M	Yousafzai/KPK/Yes	Delay in achieving milestones, abdominal distention, aggressive behavior, speech delay, hernia, short stature, coarse facial features, joint contractures, hepatosplenomegaly, low IQ, intellectual disability, dysostosis multiplex
M	VI-1	1	4.5 y/M	Rajput Bhatti, Khosa Baloch/Punjab/No	Delay in achieving milestones, abdominal distention, aggressive behavior, speech delay, short stature, constipation, coarse facial features, joint contractures, hepatosplenomegaly, intellectual disability, hernia
N	II-1	1	5 y/M	Aryn/Punjab/No	Hernia, short stature, coarse facial features

AJK, Azad Jammu and Kashmir; ESR, erythrocytes sedimentation rate; F, female; HCT, hematocrit; ICT, Islamabad capital territory; IQ, Intelligent quotient; KPK, Khyber Pakhtunkhwa; M, male; MCV, mean corpuscle volume; MCH, mean corpuscle hemoglobin; MCHC, mean corpuscle hemoglobin concentration; m, months; RBG, random blood glucose; y, years.

### 2.3 Blood collection and DNA extraction

Peripheral blood samples of 3–5 ml were taken in an EDTA vacutainer (BD vacutainer K2 EDTA 18 mg) and stored as whole blood at −20°C before DNA extraction. DNA extraction was performed using standard Phenol-Chloroform method (Grimberg et al., 1989). DNA was quantified by using the μDrop Plate reader (MultiskanTM, Thermo Fisher Scientific, and Waltham, MA, United States).

### 2.4 Whole genome sequencing and variant calling

Read mapping and genotype calling was performed using a modified version of the PALEOMIX pipeline (Schubert et al., 2014). The modified pipeline and detailed instructions are available at https://github.com/Hansen-Group/Gul2022.

Briefly, pre-analyses quality assurance was performed using FastQC v0.11.9 (https://www.bioinformatics.babraham.ac.uk/projects/fastqc/) and MultiQC v1.10 (Ewels et al., 2016). Read pairs were subsequently processed using fastp v0.20.1 (Chen et al., 2018) to trim adapter sequences, remove reads with low complexity, and merge and correct overlapping read pairs (with a minimum overlap of 11 bp). Reads were mapped using BWA mem v0.7.17-r1188 (Li, 2013). Against the hg38 human reference genome distributed as part of the GATK resource bundle (https://gatk.broadinstitute.org/hc/en-us/articles/360035890811-Resource-bundle), (McKenna et al., 2010) including alternative and decoy contigs. Mapped reads were post-processed using samtools v1.11 (Danecek et al., 2021), and the “bwa-postalt.js” script included in the BWA-kit which post-processes alignments mapping to alternative contigs such as HLA variants. Duplicate, unmapped, and orphaned reads, and secondary and supplementary alignments were filtered. The resulting BAMs were recalibrated using GATK ApplyBQSR trained using dbSNP release 151 (Sherry et al., 2001) for known variable sites.

Genotypes (GVCFs) were called for each sample using GATK HaplotypeCaller, merged, and called using GATK GenotypeGVCFs. Variant recalibration was carried out separately for SNPs and indels using GATK ApplyVQSRNode, trained using positive lists from the GATK resource bundle. Features used in the calibration were ExcessHet, DP, MQ, QD, SOR, FS, ReadPosRankSum, MQRankSum, and BaseQRankSum (only for SNPs). We kept only variables within the upper 98.0 tranche for indel and 99.6 tranche for SNPs. The resulting VCFs were annotated using VEP v104 (McLaren et al., 2016) using the Ancestral Allele, ExACpLI, GERP Conservation Scores, and LOFTEE v1.0.3 (https://github.com/konradjk/loftee) (Karczewski et al., 2020) plugins. Additional custom annotation was included as described in the github repository.

#### 2.4.1 Data analysis and variant prioritization

All variants were evaluated as per ACMG guidelines (Richards et al., 2020). Variants that were classified to be pathogenic, likely pathogenic or uncertain significance according to ACMG guidelines were prioritized.

Runs of homozygosity (ROH)-based inbreeding coefficient (F) and principal component analysis was calculated using PLINK(v1.90b6) on a dataset including both affected and unaffected individuals as well as 77 additional individuals with Pakistani origin that are part of an ongoing LSDs study (unpublished) for better allele frequency estimates. Results are only shown for the individuals in present study.

For ROH calculation, only variants with minor allele frequency MAF > 5%, and no missingness were used. ROHs were defined as having more than 100 variants, a total length larger than 1 MB, on average at least 1 variant per 50 kB, consecutive variants less than 1 MB apart, and at most 1 heterozygous call per window of 50 variants. The total length of ROHs was then divided by the total length of the genome (3 GB).

Principal component analysis was performed on by first merging with the 1000 genomes project (https://www.nature.com/articles/nature15393). Non-overlapping sites and sites with a MAF difference between LSD-samples and the 1000 genomes South Asian super population of more than 25% were removed. The PCA was calculated using PLINK--pca function on remaining overlapping sites with overall MAF > 0.05, no missingness, and LD-pruned (window size of 1 MB, step size of 1, R2-threshold of 0.8).

Non-synonymous variants including missense, non-sense, frameshift and indels in coding and splice sites changes in non-coding regions with a MAF of less than 0.01 in large, outbred populations (gnomAD exomes of South Asian population) were considered as putative causal candidates. Further prioritization of these rare variants was based on segregation with disease phenotypes within respective family. As a first phase for WGS data analysis, already reported genes causing MPS were screened for pathogenic variants and during second phase, unsolved families were checked for variants in other LSDs-related genes. In 12 families (A–L) diagnosed with MPS I, variants homozygous in affected individuals and heterozygous in their parents were prioritized based on recessive inheritance pattern of the disease and consanguinity among parents revealed by their pedigrees. The lists of heterozygous variants in each family were searched for compound heterozygous or digenic inheritance. In two families (M, N) diagnosed with MPS II, pathogenic variants on X-chromosome segregated from the mother were extracted. In families where genotypes of parents were available, exomes of each family were analyzed for dual molecular diagnosis (Supplementary Figure S1). As a reference population, more than a 100 healthy control samples from the general Pakistani population were analyzed for the prevalence of potentially causal variant as per ACMG guidelines.

The identified variants were subjected to *in silico* analysis using the following tools: Have (y) Our Protein Explained (HOPE) (https://www3.cmbi.umcn.nl/hope/), Predictor of human deleterious single nucleotide polymorphisms (PhD-SNP) (https://snps.biofold.org/phd-snp/phd-snp.html), Protein analysis through evolutionary relationships (PANTHER) (http://www.pantherdb.org/tools/csnpScoreForm.jsp), I-Mutant (https://folding.biofold.org/i-mutant/i-mutant2.0.html), MUpro (http://mupro.proteomics.ics.uci.edu/), VarSome (https://varsome.com/), and ACMG classification (http://wintervar.wglab.org/).

## 3 Results

### 3.1 Clinical characterization

In total, fourteen families (A-N) of Pakistani origin with phenotypes associated with MPS I and MPS II were included in the present study. Patients showed a wide variety of characteristic symptoms from both MPS I and MPS II including coarse facial features, joint contractures, abdominal distention, hepatosplenomegaly, hernia, and mental abnormalities among others (Table 1). Twelve of fourteen (A-L) families showed consanguinity and clinical phenotypes consistent with MPS I and were consequently labeled as suspected cases of MPS I by the clinicians (Table 1; Figure 1). The other two families (M and N) did not show consanguinity and phenotypes matched clinically with MPS II (Table 1; Figure 2).

### 3.2 Genetic characterization

**FIGURE 4:**
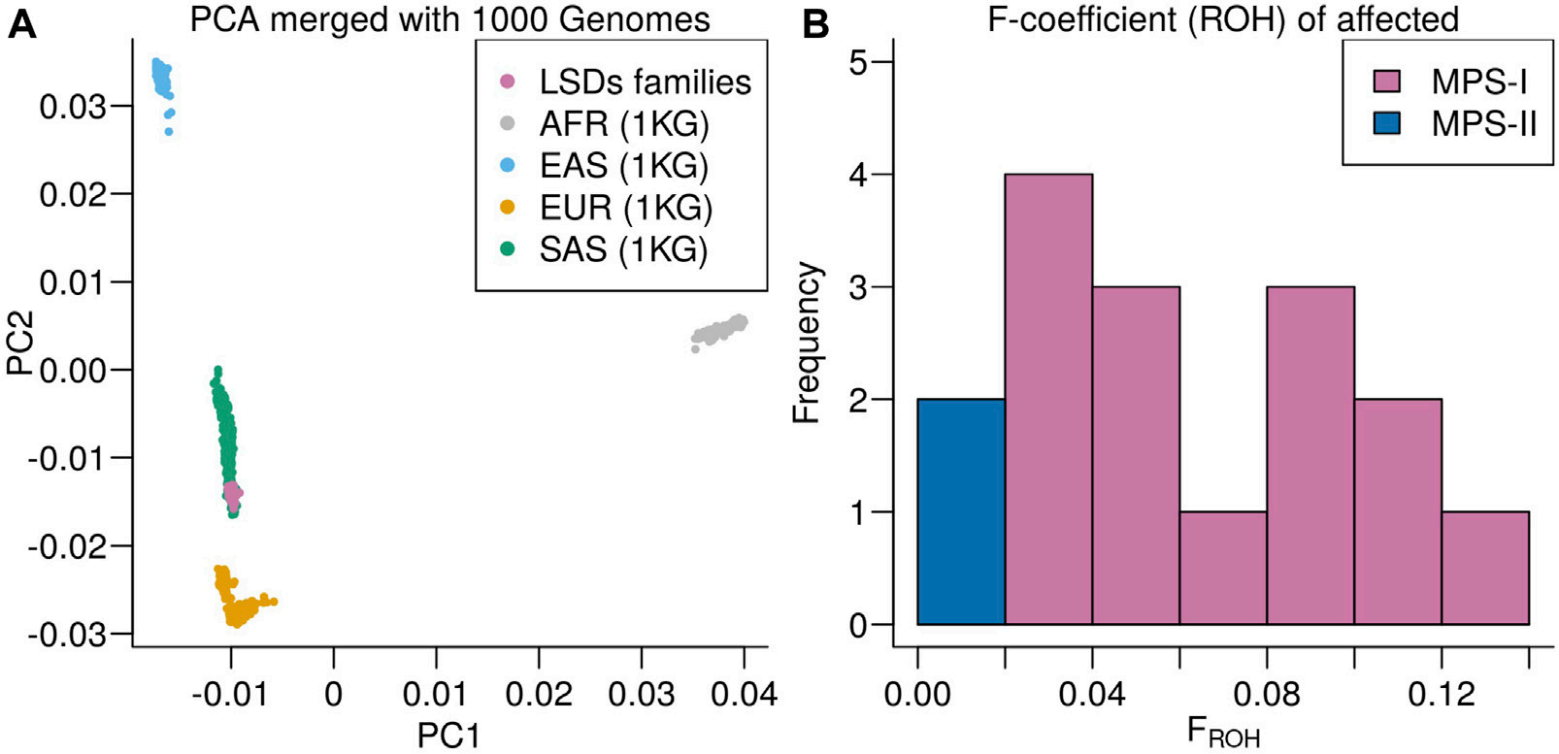
**(A)** Principal component analysis of the LSDs families merged with the 1000 Genomes project colored according to super population: African ancestry (AFR), East Asian ancestry (EAS), European ancestry (EUR), and South Asian ancestry (SAS). **(B)** Distribution of runs of homozygosity-based F-coefficient of the LSDs families.

Principal component analysis of the LSDs families merged with the 1000 genomes projects showed the LSDs families clustering with the South Asian (SAS) superpopulation (Figure 4A). The SAS population includes a Punjabi population (PJL) verifying the genetic ancestry of the LSDs families. Runs of homozygosity-based F-coefficient of the affected individuals showed that the high rate of consanguinity in MPS I families was also reflected in the genetic data (Figure 4B) and close to 0 for the non-consanguineous MPS II affected individuals.

Analysis of WGS data of available affected and unaffected individuals in each family revealed ten novel and six previously reported homozygous or hemizygous variants in the genes involved in MPS I, MPS II or other lysosomal storage disorders. Five families (C, I, K, J, L) showed multigenic inheritance (Table 2). All the variants were found in ROH in homozygous/hemizygous state in affected individuals while heterozygous or wild type in their parents. In family D, no homozygous or compound heterozygous variants were found in MPS-related genes. Therefore, screening for other LSD-associated or novel candidate genes was performed. The analysis revealed a homozygous missense variant (c.26569G>A; R857C) in *ABCA5* located in the ROH. Based on the gene expression, function and pathways, *ABCA5* was predicted to be the strongest candidate. Phenotypically normal siblings were either wild type or heterozygous carrier of the respective variants in each family (Figures 1, 2). Genes were annotated according to the following transcripts: NM_000512.5 (GALNS); NM_000203.5 (IDUA); NM_000199.5 (SGSH); NM_000152.5 (GAA); NM_172232.4 (ABCA5); NM_001166550.4 (IDS) NM_000035.4 (ALDOB); NM_001384125.1 (KIAA1109), NM_016146.6 (TRAPPC4); NM_003922.4 (HERC1); NM_139125.4 (MASP1); NM_014140.4; NM_012250.6 (RRAS2).

**TABLE 2:** List of variants identified in the current study.

Pedigree/Patient ID	Gene variant	Patients’ genotype	dbSNP	*In-silico* tools with deleterious output	MAF gnomAD	Classification ACMG	VarSome	HGMD accession number
A/V-4	*GALNS*	Homozygous	rs118204448	4/7	0.00001972	Pathogenic	Pathogenic	CM950535
	NM_000512.5:c.871G>A (p.Ala291Thr)					PP3, PP4, PP5, PM1, PM2		
B/V-1	*IDUA*	Homozygous	rs1560548356	2/4	0.000007	Likely pathogenic	Likely pathogenic	Current study
	NM_000203.5:c.1172-1173insGCTGCTGGC (p.Gua391insLeuLeuAla)					PP4, PM2, PM4		
C/V-1	*SGSH*	Homozygous	rs104894636	17/17		Pathogenic	Pathogenic	CM971355
	NM_000199.5:c.220G>A (p.Arg74Cys)				0.0002270	PP5, PS3, PM1, PM2, PM5, PP3		
	*GAA*		rs752735168	16/18	0.00003306	Likely Pathogenic	Pathogenic	Current study
	NM_000152.5:c.1930G>T (p.Ala644Ser)					PM1, PM2, PP3, PP4		
D/IV-1	*ABCA5*	Homozygous	rs747667004	7/7	0.0001090	Likely Pathogenic	Uncertain significance	Current study
	NM_172232.4:c.26569G>A (p.Arg857Cys)					PM1, PM2, PP3, PP4		
E/IV-2, IV-4	*IDUA*	Homozygous	rs121965027	7/7	0.00004681	Pathogenic	Pathogenic	CM950685
	NM_000203.5:c.1469T>C (p.Leu490Pro)					PS3, PM1, PM2, PP4, PP5, PS3		
F/IV-1	*IDUA*	Homozygous	rs121965027	7/7	0.00004681	Pathogenic	Pathogenic	CM950685
	NM_000203.5:c.1469T>C (p.Leu490Pro)					PS3, PM1, PM2, PP4, PP5, PS3		
G/IV-4	*IDS*	Hemizygous	—	4/4	—	Pathogenic	Pathogenic	Current study
	NM_001166550.4:c.353T>A (p.Leu118Ter)					PVS1, PM2, PP3, PP4		
H/IV-2	*ALDOB*	Homozygous	rs200585150	11/18	0.0004219	VUS	Uncertain Significance	CM1618755
	NM_000035.4:c.264G>T (p.Asp88Glu)					PM1, PM2, PP3 BP1		
I/V-1	*IDUA*	Homozygous	rs121965027	7/7		Likely Pathogenic	Pathogenic	CM950685
	NM_000203.5:c.1469T>C (p.Leu490Pro)				0.00004681	PS3, PM1, PM2, PP4, PP5		
	*KIAA1109*		rs773658128	6/7	0.00001213	VUS	Uncertain significance	Current study
	NM_001384125.1:c.1297A>G (p.Thr4033Ala)					PM1, PM2		
J/V-2	*TRAPPC4*	Homozygous	rs375776811	N/A	0.0002403	Pathogenic	Likely pathogenic	Not available
	NM_016146.6:c.454 + 3A>G				-	PP5, PM2, PP3		
	*HERC1*		—	4/7		VUS	Uncertain significance	Current study
	NM_003922.4:c.5941G>A (p.Val1981Ile)					PM2, BP4		
K/IV-4	*IDUA*	Homozygous	rs121965027	7/7	0.00004681	Likely Pathogenic	Pathogenic	CM950685
	NM_000203.5:c.1469T>C (p.Leu490Pro)				0.0001591	PS3, PM1, PM2, PP4, PP5		
	*MASP1*		rs369042881	N/A		VUS	Uncertain significance	Current study
	NM_139125.4:c.C73T (p.Arg25Ter)					PM2, BS1, BS2		
L/IV-5	*SMARCAL1*	Homozygous	—	6/7	-	Pathogenic	Likely pathogenic	Current study
	NM_014140.4:c.1810C>T (p.Gln604Ter)				-	PVS1, PM2, PP3		
	*RRAS2*		—	16/18		Likely Pathogenic	Likely Pathogenic	Not available in HGMD
	NM_012250.6:c.439C>T (p.Arg147Trp)					PM1, PM2, PP2, PP3		
M/VI-1	*IDS*	Hemizygous	—	17/23	—	Likely Pathogenic	Likely pathogenic	Current study
	NM_000202.8:c.1264C>G (p.Cys422Ser)					PM1, PM2, PP3, PP4, BP1		
N/III-1	*IDS*	Hemizygous	—	11/11	—	Pathogenic	Pathogenic	Current study
	NM_000202.8:c.1035C>T (p.Trp345Ter)					PVS1, PM2, PP3		

N/A, not applicable; VUS, variant of uncertain significance; -, not found.

These variants were predicted pathogenic, likely pathogenic or variant of uncertain significance according to ACMG classification (Table 2). None of the identified variants was found in homozygous state in any human genome variations databases including gnomAD, ExAC, 1000 Genome Project and ethnically matched healthy controls.

## 4 Discussion

In the present study, fourteen families (A-N) initially diagnosed with MPS underwent WGS to identify potentially pathogenic variants. Among these, twelve families (A-L) were clinically characterized as MPS I while two (M-N) were clinically diagnosed as MPS II. The diagnosis was based on clinical phenotypes including facial features, stature, laboratory tests and inheritance of the phenotypes in pedigree. WGS revealed homozygous variants in previously MPS-associated genes (*IDUA, GALNS, SGSH, GAA* and *IDS*) and a novel LSDs-associated candidate gene (*ABCA5*) in affected individuals. In addition to these genes, variants were found in seven genes (*ALDOB, MASP1, KIAA1109, TRAPPC4, SMARCAL1, HERC1, RRAS2*) that were previously not reported in MPS but associated with other metabolic disorders.

Our findings in family A (i.e., MPS I), reported a homozygous sequence variant (c.871G>A; A291T) in *GALNS.* The gene encodes N-acetylgalactosamine-6-sulfatase which is a lysosomal exohydrolase required for the degradation of the glycosaminoglycans, keratan sulfate, and chondroitin 6-sulfate. Previously, the identified variant (A291T) has been reported in compound heterozygous and homozygous forms in two different families of Pakistani origin segregating MPS IV (Tomatsu et al., 1995; Ullah et al., 2017). *In-silico* analysis revealed that missense mutation (p.A291S) resulted in the formation of additional intramolecular interactions, most likely affecting the conformation of the active site (Asp288 and Asn289) and its substrate binding ability. Therefore, variation at amino acid position 291 of GALNS would affect structure and enzymatic function of the protein.

Multiple mutations in *IDUA* gene have been identified and associated with MPSI (Tieu et al., 1995). The *IDUA* gene is located at 4p16.3 and comprises 14 exons. IDUA codes for the enzyme α -L-iduronidase, which is required for the degradation of the GAGs dermatan and heparan sulfate. Deficiency of the enzyme leads to the accumulation of these GAGs in tissues throughout the body, playing a central role in the pathogenesis of MPS I. A novel homozygous insertion mutation (c.1172-1173insGCTGCTGGC; G391insLLA) in *IDUA* was identified in an affected individual in family B. Glycine at position 391 is involved in the formation of torsion angles due to its high degree of rotational ability which maybe lost after the insertion of LLA leading to abnormal function of the enzyme. Furthermore, affected individuals in families E, F, I and K also revealed a previously reported variant (c.1469T>C; L490P) in *IDUA* (Gul et al., 2020). In these cases, a high interfamilial variability in the phenotypes of patients segregating same sequence variant (*IDUA*; c.1469T>C; L490P) was observed in our families (E, F, I, K). Deep analysis of the genome of individuals revealed additional homozygous pathogenic variants in other genes (c.1297A>G; T4033A in *KIAA1109* in family I, and c.C73T; R25* in *MASP1* in family K) that may act as modifiers or dual molecular diagnosis. Previously sequence variants in *KIAA1109* (4q27) encoding a protein playing role in endosomal trafficking and endosome recycling of lipids have been reported to cause Alkuraya-Kucinskas syndrome characterized by brain abnormalities associated with cerebral parenchymal underdevelopment, arthrogryposis, clubfoot, and global developmental delay. Patients in family I showed phenotypes of MPS as well as Alkuraya syndrome. Similarly, sequence variants in *MASP1* (3q27.3) encoding a serine protease having an essential role in the innate and adaptive immune response have been associated with 3MC syndrome 1 characterized by widely spaced eyes (hypertelorism), a narrowing of the eye opening (blepharophimosis), droopy eyelids (ptosis) and highly arched eyebrows. Patients in the present family K showed phenotypes of 3MC syndrome in addition to MPS.

*IDUA*, *KIAA1109* and *MAPS1* are located at different loci of human genome. Due to higher rate of consanguinity, the number of homozygous regions increases leading to a high risk of occurrence of multiple pathogenic variants. This might lead to complex phenotypes due to co-segregation of two syndromes in the same patient. Therefore, analyzing genomes of populations with a high rate of consanguinity, dual molecular diagnosis should be considered.

In family C, we can appreciate a multi-genic inheritance, segregating two damaging variants in known LSDs enzymes SGSH (c.220G>A; R74C) and GAA (c.1930G>T; A644S) in homozygous state, both of which lie within the mapped interval at 17q25.3. *SGSH* encodes a lysosomal enzyme sulfamidase involved in the lysosomal degradation of heparan sulfate. Pathogenic sequence variants in the gene lead to MPS IIIA (Weber et al., 1997). Arginine at amino acid position 74 is likely to be involved in the formation of the active site of sulfamidase. Transition of arginine at the position to a non-conserved Cysteine may grossly affect enzyme activity. *GAA* encodes a lysosomal enzyme alpha-glucosidase. The lysosomal alpha-glucosidase is essential for the degradation of glycogen to glucose in lysosomes. Defects in the enzyme lead to glycogen storage disease. In addition to MPS phenotypes associated with variants in *SGSH*, the patient showed low hemoglobin concentration which is found in glycogen storage disease caused by pathogenic variant in *GAA*. These findings, suggest that the disease phenotypes in the present patient could be due to the contribution of variants in both genes, *SGSH* and *GAA*.

In family D, we have identified a missense homozygous variant (c.26569G>A; R857C) in *ABCA5.* The ABCA5 protein is a member of the superfamily of ATP binding cassette (ABC) transporters. ABC proteins play an important role in transporting various molecules across extra- and intracellular membranes. Loss of function variants in human *ABCA5* have been associated with hair overgrowth in human (DeStefano et al., 2014; Raza et al., 2020). On the other side, *abca5−/−* knockout mice developed lysosomal disease-like symptoms (Kubo et al., 2005). Interestingly, the affected individual in family D carrying *ABCA5* missense variant showed phenotypes overlying with MPS associated with hair overgrowth. To the best of our knowledge, this is the first human case associating a variation in *ABCA5* with LSD associated with hair overgrowth.

ABCA5 has 1642 amino acids long lysosomal peptide which is composed of two domains including ABC transporter 1 (478-713) and ABC transporter 2 (1290-1533). The protein determines the fate of cholesterol derived from lipoprotein. The functional loss of this protein impairs the integrity of lysosomes, disrupts the intracellular flow of free cholesterol, and causes cholesterol to accumulate intra-endo-lysosomally, adding to the organelles’ malfunction (Raza et al., 2020). The substitution R857C, replaces a positively charged, less hydrophobic bigger amino acid (Arginine) to a neutral, more hydrophobic and smaller amino acid (Cysteine). Change in charge, size and nature of amino acids (Arginine and Cysteine) at position 857 may affect interactions of the protein with other molecules and residues leading to improper protein folding and intra-lysosomal accumulation of cholesterol.

In family G, a homozygous non-sense variant c.353T>A; p.L118* was identified in *IDS* gene which is involved in the X-linked inheritance of MPS II. IDS gene encodes a protein iduronate 2-sulfatase that catalyzes the degradation of heparan sulfate and dermatan sulfate (Gul et al., 2020).

In family H, a homozygous missense variant (c.264G>T; p.D88E) was identified in *ALDOB*. *ALDOB* encodes fructose-1,6-bisphosphate aldolase, a tetrameric glycolytic enzyme that catalyzes the reversible conversion of fructose-1,6-bisphosphate to glyceraldehyde 3-phosphate and dihydroxyacetone phosphate. Pathogenic variants in the gene have been associated with fructose intolerance. The specific variant (c.264G>T) identified in family H was previously reported by Retterer et al. (2016) in a family segregating abnormality of central nervous system. Interestingly, the affected individual in our family H, showed delay in achieving milestones, abdominal distention, coarse facial features, aggressive behavior, short stature, respiratory/ear infections, hip dysplasia, joint stiffness, hepatosplenomegaly, difficulty in walking, craniofacial disproportion, J-shaped sella, L1 vertebral body is hypoplastic and slightly posteriorly displaced and exaggerated lumbar lordosis. In addition to these phenotypes, the patient showed hypoglycemia, which is an indication of fructosuria. The amino acid aspartic acid D at position 88 make salt bridge with R57, L92 and L321, the mutant residue glutamic acid E may not be able to interact. Wild type residue D is in its preferred secondary structure, a turn, while mutant residue E prefers to be in another secondary structure, which may therefore destabilize the protein conformation. Due to mutated fructose-1,6-bisphosphate aldolase, accumulation of fructose-1,6-bisphosphate in different organs of the body may lead to multi-organ abnormality.

Family J revealed two homozygous variants, one is a missense variant c.5941G>A; p.V1981I in *HERC1* gene and other is splice site variant (c.454 + 3A>G) in *TRAPPC4*. HERC1 protein is involved in the membrane trafficking *via* guanine nucleotide exchange factors (GEF) and previously reported as a novel candidate gene for causing intellectual disability (Ortega-Recalde et al., 2015). Recently, Van Bergen and colleagues (Van Bergen et al., 2020) reported the same splice variant underlying early-onset seizures, developmental delay, microcephaly, sensorineural deafness, spastic quadriparesis and progressive cortical and cerebellar atrophy in families of Caucasian, Turkish and French-Canadian ethnicities. Other authors also identified the same splice site variant in a family of Indian origin having progressive encephalopathy and muscle involvement (Kaur et al., 2020). The primary phenotypes of our patient in family J include delay in achieving milestones, aggressive behavior with multiple crying episodes, respiratory/ear infection, unable to walk, and intellectual disability. Interestingly, phenotypes like muscles weakness, deafness, microcephaly and seizures were not found in our case. The phenotypic variability might be due to different familial backgrounds of the families and effect of rare SNPs in modifier genes. The variant is predicted to cause frameshift of the protein due to mis-splicing of mRNA.

In affected individual of family L, a novel homozygous missense variant c.439C>T; p.R147W in *RRAS2* gene and a novel non-sense variant (c.1810C>T; Q604*) was found in *SMARCAL1*. The substitution in gene *RRAS2* of a positively charged arginine at position 147 with a neutral residue tryptophan may lead to the loss of ionic interactions with surrounding residues. It also leads to distortion of shape as mutant residue tryptophan is bigger in size than wild type residue arginine. RRAS2 protein is involved in the regulation of MAPK signaling pathway (Capri et al., 2019). The involvement of *RRAS2* gene in some disease is still to be explored and linked with disease etiology in human genome mutation database. The encoded protein SMARCAL1 is a member of the SWI/SNF family of proteins. Members of this family have helicase and ATPase activities and are thought to regulate transcription of certain genes by altering the chromatin structure around those genes. Pathogenic variants in the gene have been reported in nephrotic syndrome and a metabolic syndrome Schimke immuno-osseous dysplasia characterized by short stature, kidney disease, and a weakened immune system. The patient in the present study carrying a non-sense variant in the gene showed a severe phenotype of delay in achieving milestones, abdominal distention, aggressive behavior, speech delay, hernia, short stature, coarse facial features, joint contractures, hepatosplenomegaly, intellectual disability and dysostosis multiplex (Table 1). Neurological phenotypes including intellectual disability, aggressive behavior, joint contractures, and hepatosplenomegaly showed by the present case were not described in previous cases. The variation in the phenotypes of previous and the present case might be due to the nature and/or position of mutation, different familial background and ages of the affected individuals. The identified non-sense variant (Q604*) in SMARCAL1 is predicted to cause loss of function of the protein either through non-sense-mediated mRNA decay or production of truncated protein.

Regarding families diagnosed as MPS II (M and N) we found pathogenic variants (c.1264C>G; C422S in family M and c.1035C>T; W345* in family N) in *IDS* segregating with the phenotypes. The novel missense variant C422S affects a non-conserved residue among human sulfatases. Cysteine is more hydrophobic in nature than serine, which causes loss of hydrophobic interactions. Cysteine at position 422 is involved in the sulphide bridge formation which will be lost after this change with serine thus it will affect the stability of protein structure and ultimately loss of function. The other non-sense variant (W345*) in IDS was previously identified in Japanese patient affected with sever phenotypes of MPS II (Sukegawa et al., 1995). The authors found a smaller precursor protein in cells transfected with W345*. Therefore, it is predicted that the variant leads to the production of truncated enzyme. The affected individual in family N showed mild phenotypes of short stature, coarse facial features and hernia as compared to those reported by Sukegawa et al. (1995).

Our study increases the knowledge of genetic factors involved in rare diseases. For this purpose, the Pakistani population is unique due to the high number of families with consanguineous marriages and the high frequency of large pedigrees. Furthermore, we have used WGS instead of phenotype-based diagnosis to diagnose subtypes of disorders having overlapping phenotypes. Thus, WGS is a fruitful strategy to identify novel causal homozygous variants in inbred populations.

The findings of this study have to be taken with some limitations. For example, limited clinical details were gathered from the recruited families due to limited resources like testing facilities at tertiary care hospital at the time of recruitment of the patients. The overlapping phenotypes among the recruited cases presented in this study is one of the main reasons for pseudo-identification of various pathologies diagnosed initially as MPS. After getting results from sequencing data, the families were recontacted for more detailed evaluation but most of the recruited patients have died and families flatly declined to provide more information. Thus, the present study could not include functional enzymatic studies and comprehensive analysis of 3D protein structure. Performing *in-vitro* and *in-vivo* functional studies would help in examining detailed biological effects of the identified variants. Further studies including more consanguineous families with similar phenotypes and combined with functional analysis should be performed to clarify the effect of the identified variants.

Genetic findings in the affected families showed that the diagnosis based on phenotypic presentation is not an optimal method. For this reason, the incorporation of next-generation sequencing (NGS) in consanguineous families represents one of the best ways to elucidate the underlying causes in rare diseases such as MPS I and MPS II and establish a genotype-phenotype relationship (Hoffman et al., 2013; Umair et al., 2018). NGS will not only be helpful in the correct diagnosis of the disease but it will also help in prenatal testing, family planning, carrier testing and genetic counseling in families affected with LSDs (Komlosi et al., 2016). Similarly, dual molecular diagnosis has been reported in several cases of consanguineous unions (Umair et al., 2017), which cannot be detected through conventional sequencing or enzymatic testing. Therefore, NGS is required to identify all the pathogenic and modifier variants in heterogeneous disorders like LSDs. Sequencing highly homologous regions (for example, *GBA1* locus) using Sanger or short-read NGS methods in suspected cases of LSDs can miss other mutant alleles. Because, the shorter reads cannot be mapped uniquely to the reference genome, especially in cases where there are recombinant alleles aligning to the homologous region. Therefore, long-read NGS is recommended in such cases to discriminate the functional genes from their pseudogenes (Woo et al., 2021).

Accurate and early diagnosis of LSDs in children represents a helpful step for designing therapeutic strategies to save different organs from permanent degeneration. Pre-natal screening and identification of carriers’ status in an affected family for LSDs will be helpful for genetic counselling of the family. The identification of a novel MPS I gene (*ABCA5*) based on WGS proves to be helpful for expanding the limits of targeted therapies to treat LSDs.

## Data Availability

The data presented in the study are deposited in the ClinVar repository, accession numbers: SCV003842213 (*IDS*:c.1035G>T); SCV003842212 (*IDS*:c.1264T>G); SCV003842211 (*MASP1*:c.73A>T); SCV003842210 (*KIAA1109*:c.1297C>G); SCV003842209 (*ABCA5*:c.2569C>T); SCV003842208 (*SMARCAL1*:c.1810C>T); SCV003842207 (*HERC1*:c.5941G>A); SCV003842206 (*IDS*:c.353T>A); SCV003842205 (*GAA*:c.1930G>T); SCV003844061 (*RRAS2*:c.439C>T).

## Supplementary material

SUPPLEMENTARY FIGURE S1 Schematic diagram of the study showing recruitment and clinical diagnosis of affected families followed by whole genome sequencing and variants prioritization.
