# Supplementary figures and images for: Identifying the genetic causes of phenotypically diagnosed Pakistani mucopolysaccharidoses patients by whole genome sequencing
Source: Front Genet. 2023 Apr 5;14:1128850. doi: 10.3389/fgene.2023.1128850 (PMC10113632; doi:10.3389/fgene.2023.1128850)

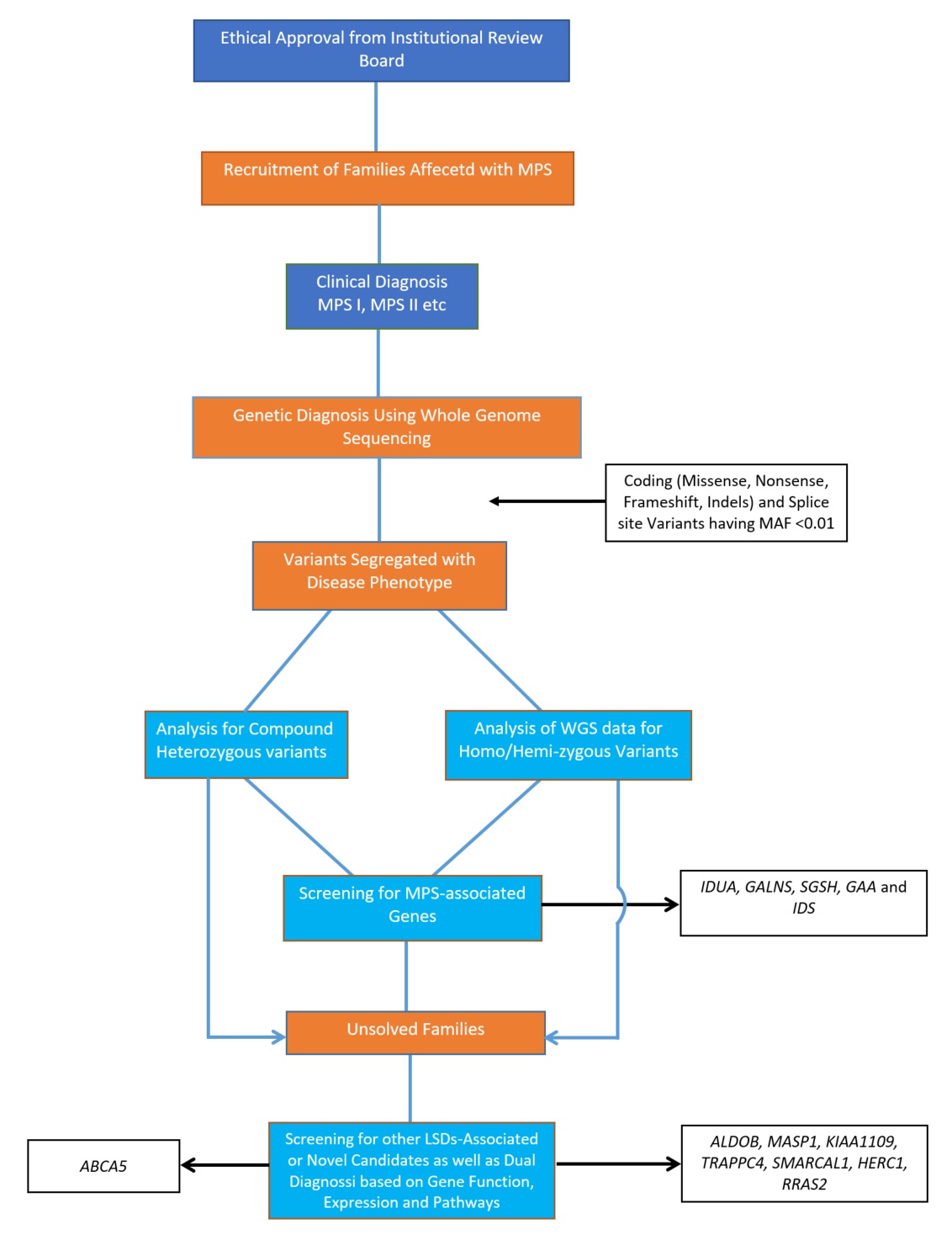

Supplement: Supplementary file 1 [file Image1.jpeg]
